# Lonely Individuals Do Not Show Interpersonal Self-Positivity Bias: Evidence From N400

**DOI:** 10.3389/fpsyg.2018.00473

**Published:** 2018-04-06

**Authors:** Min Zhu, Changzheng Zhu, Xiangping Gao, Junlong Luo

**Affiliations:** ^1^Department of Social Work and Management, Nanjing Tech University, Nanjing, China; ^2^Institute of Social Innovation and Development, Nanjing Tech University, Nanjing, China; ^3^Department of Psychology, Shanghai Normal University, Shanghai, China; ^4^Department of Psychology, Henan University, Kaifeng, China; ^5^Academic Affairs Office, Shanghai Normal University, Shanghai, China

**Keywords:** interpersonal self-positivity bias, social interaction, ERP, N400, loneliness

## Abstract

Self-positivity bias is one of the well-studied psychological phenomena, however, little is known about the bias in the specific dimension on social interaction, which we called herein interpersonal self-positivity bias—people tend to evaluate themselves more positively on social interactions, prefer to be included rather than to be excluded by others. In the present study, we used a modified self-reference task associated with N400 to verify such bias and explore whether impoverished social interaction (loneliness) could modulate it. Findings showed that exclusion verbs elicited larger N400 amplitudes than inclusion verbs, suggesting that most people have interpersonal self-positivity bias. However, loneliness was significantly correlated with N400 effect, showing those with high scores of loneliness had smaller differences in the N400 than those with lower scores. These findings indicated impoverished social interaction weakens interpersonal self-positivity bias; however, the underlying mechanisms need to be explored in future research.

“The self-construction process is intrinsically rooted within, and dependent upon, interpersonal processes that unfold in the social world.”

Morf and Mischel, 2012

## Introduction

Self is fundamentally interpersonal (Morf and Mischel, 2012). Interactions with others influence one’s sense of self-esteem, self-worth, which plays a quite important role in the definition of sense of self (Richman et al., 2016). Having a good relationship with people around will fulfill one’s need of belonging and connection, which is implicitly constructed into individual’s self-concept. On the contrary, social exclusion described as social death to human beings (Williams, 2007) makes people feel distressed and painful, which has a negative influence on the sense of self. Thus, people tend to maintain positivity about themselves on social interactions rather than be isolated or excluded, which we called herein “interpersonal self-positivity bias.” The aim of the present study is to examine this bias by exploring how people process inclusion and exclusion cues at neural level.

Humans hold positive views about themselves, which is robust and cannot be modulated by education (Myers, 2010). For example, people speak highly of their ability (Gawande, 2002); evaluate themselves more positively (Heine et al., 1999; Watson et al., 2007) and attribute good things to themselves (Mezulis et al., 2004). These phenomena are well-studied as self-positivity bias indicating that information on self is of positive valance. Previous studies on self-positivity bias mainly focus on traits by using the emotional trait adjectives as stimuli (Herbert et al., 2006, 2008; Kiefer et al., 2007) and the self-referential judgments task (e.g., Zhou et al., 2013; Chen et al., 2014; Metzler et al., 2014; Kiang et al., 2017), for example “I am careful” or “I careful” (primes are pronouns, targets are trait adjectives). Self-positivity bias is reflected by the discrepancy that people response faster to self-positive words and non-self-negative words than to self-negative and none-self-positive words. Healthy people endorse more positive personality traits as more congruent with their self-concept than negative traits (Herbert et al., 2011b). Specifically, self is an interpersonal self-construction system (Morf and Mischel, 2012), by interacting with others, we define who we are, with what kind of traits. Therefore, status of interpersonal relationship influences self-positivity bias specific on interpersonal dimension. People hold the positive interpersonal bias about themselves that they are liked, included, and accepted by other people. Experience of being excluded weakens individuals’ memory of self-related information (Hess and Pickett, 2010), leading individuals to avoid self-awareness (Twenge et al., 2003). However, little empirical research was conducted to confirm this interpersonal self-positivity bias. Thus, we presumed that people generally have this significant interpersonal bias, which is specific of, but different from general self-positivity bias.

Social interaction plays quite an important role in self-concept creating and maintaining, on the contrary, the lack of social interaction with others will do harm to people’ knowledge of self. Loneliness is defined as a psychological state resulting from dissatisfaction with the number and quality of one’s social relationships (Goswick and Jones, 1981). People who have a high score of sense of loneliness are chronically in impoverished social interaction situations and always feel disconnected with others (Richman et al., 2016). Goswick and Jones (1981) investigated the relationship between loneliness and self-concept, and found loneliness was significantly negatively related to social self, personal self and physical self, but not related to family self, moral-ethical self, and self-criticism. Otherwise, Lack of meaningful social interaction makes lonely people self-confusion (Richman et al., 2016). So far we learned little about what changes to interpersonal self-positivity bias may take place along with an increase of sense of loneliness. Studies on attribution indicated that lonely people, no matter children (Crick and Ladd, 1993; Renshaw and Brown, 1993) or adults (Anderson et al., 1983), are easily trapped into a self-defeating attribution pattern in which they attribute social failure to stable and internal factors. For example, lonely people are more likely to attribute social exclusion to internal factors, such as their lack of likability (Vanhalst et al., 2015). In addition, lonely individuals are prone to have lower expectancies to obtain future relationship, tend to have less positive response to social inclusion (Moller et al., 2010; Vanhalst et al., 2015). An fMRI study also found that lonely people were different from non-lonely people in activation of ventral striatum, showing less rewarded by positive social stimuli (people pictures) (Cacioppo et al., 2009). Therefore, we presumed that loneliness would impair interpersonal self-positivity bias.

N400 is a negative-going deflection occurring 200–600 ms (peaking around 400 ms) after meaningful stimulus onset, largest over centro-parietal sites, with a slightly right hemisphere bias (at least evidenced by written words in sentences) (Kutas and Federmeier, 2011). It is a useful component reflecting processes associated with integrating semantic information into the context or a mental representation. Study showed that negative stereotypes about rural migrant workers (RMWs) lead larger N400 when positive adjectives paired with RMWs compared with positive adjectives paired with urban workers (Wang et al., 2011). Racial stereotype research found that greater N400 was elicited when primes and traits mismatched (Hehman et al., 2014). In the self-reference task, N400 amplitude is viewed as an important index of mismatched extent between traits and positive self-concept (Watson et al., 2007; Zhou et al., 2013; Chen et al., 2014; Metzler et al., 2014; Kiang et al., 2017). Self-positive adjectives elicited smaller N400 than self-negative adjectives or other-positive adjectives (e.g., Watson et al., 2007; Zhou et al., 2013; Chen et al., 2014)—such N400 difference is also called the N400 effect. If people have a negative self-concept (e.g., people in depression) or a disturbed self-concept (e.g., schizophrenia patients), the N400 effect is smaller (Metzler et al., 2014; Kiang et al., 2017).

In the present study, we investigated interpersonal self-positivity bias by a modified self-reference task with N400. In the modified reference task, participants were instructed to read passive sentences, the primes were “I” and “am” and the endings of sentences were verbs. Semantic meanings of these verbs reflected situation of interpersonal relationship with others, dividing into inclusion condition and exclusion condition. Because being excluded violates people’s positive interpersonal self-concept, we hypothesized that exclusion verbs elicited larger N400 amplitudes than inclusion verbs. As mentioned above, lonely individuals are hyposensitive to inclusion information and hold negative self-concept which is not violated by exclusion verbs, we hypothesized that interpersonal self-positivity bias would disappear—the N400 effect may be less or not pronounced with increment of loneliness. However, lonely people might have stronger associations between self-concept and exclusion encounter, and/or weaker associations between self-concept and inclusion encounter, thus we would be cautious to speculate how N400 elicited by exclusion verbs and inclusion verbs interact with loneliness.

## Materials and Methods

### Participants

Thirty-one undergraduate and graduate Chinese students (22 females) from Shanghai Normal University participated in the experiment. Their age ranged between 18 and 24 years (mean = 19.58, *SD* = 1.69) and they were all right-handed. Participants gave written informed consent before taking part in the experiment. This study was approved by the local ethics committee of Shanghai Normal University.

### Measures

#### Self-Report Questionnaires

Participants’ sense of loneliness was measured by using the University of California, Los Angeles Loneliness scale (UCLA; Russell, 1996). The scale consisted of 20 items (e.g., ‘how often do you feel alone,’ ‘how often do you feel left out’) on four-point scale (1 = never, 2 = rarely, 3 = sometimes, 4 = often). Higher scores indicate higher levels of loneliness. In the current study, the scores ranged from 22 to 66. The scale exhibited high internal consistency, α = 0.889.

To measure levels of self-esteem, participants were also instructed to complete the Rosenberg Self-Esteem Scale (Rosenberg, 1965). The scale comprises 10 items on a four-point scale. Higher scores indicate higher levels of self-esteem. In the current study, the scores ranged from 21 to 37. Internal consistency of the scale was high (α = 0.814).

#### Stimuli

Verbs consisted of two words (37 for inclusion and 37 for exclusion) were originally collected from internet and verb database of Chinese affective words system (Wang et al., 2008). Then 17 college students were asked to rate these words on three dimensions: (1) degree of exclusion, ranging from 0 (not at all) to 3 (extremely); (2) emotional valance, ranging from 1 (extremely unpleasant) to 7 (extremely pleasant); (3) emotional intensity, ranging from 1 (extremely weak) to 7 (extremely strong). Mean ratings were computed for each of the rating dimensions, for each type and for each participant. The criterion to select was that mean rating of inclusion word was lower than 1, and mean rating of exclusion word was higher than 1. According to these criterions, 20 verbs for each type were selected (e.g., inclusion verb “accept,” “like”; exclusion verb “reject,” “ignore”; see all the words in Supplementary Material). The exclusion verbs were of significantly higher degree of exclusion [*M*_inclusion_ = 0.01 (*SD* = 0.02), *M*_exclusion_ = 1.93 (*SD* = 0.54); *t*(19.06) = 15.73, *p* < 0.001], more negative than inclusion verbs [emotional valance: *M*_inclusion_ = 5.56 (*SD* = 0.27); *M*_exclusion_ = 2.29 (*SD* = 0.49); *t*(29.45) = 27.87, *p* < 0.001], however, there’s no significant difference on emotional intensity [*M*_inclusion_ = 5.06 (*SD* = 0.33), *M*_exclusion_ = 4.99 (*SD* = 0.67); *t*(27.64) = 0.46, *p* = 0.65].

### Procedure

A few weeks before the experiment, participants completed the questionnaires.

On arrival at the experiment room, participants were instructed to carefully watch words stimuli showed on the screen. In each trial, word “I” (“
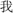
” in Chinese; visual angle, 3.15° × 2.48°) appeared on the screen; 500 ms later, a word “am” (“
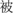
” in Chinese; visual angle, 3.15° × 2.48°) was presented which lasted for 1000 ms. Finally, the verbs (e.g., “excluded,” “
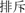
” in Chinese) measuring 6° × 2.48° in visual angle appeared for 1000 ms. All words were displayed in white on a black background. There were two blocks; one for exclusion condition (20 verbs on exclusion) and one for inclusion condition (20 verbs on inclusion), and the sequence of blocks were counterbalanced across participants. The experiment was programmed using E-Prime 2.0 (Psychology Software Tools, Inc., Pittsburgh, PA, United States).

### EEG Recording and Analysis

**FIGURE 1:**
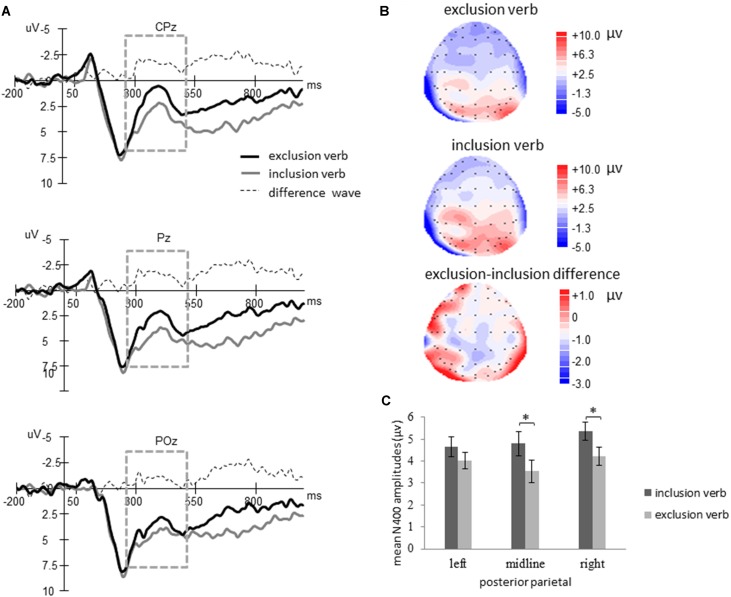
Results for N400. **(A)** Grand mean waveforms for exclusion, inclusion verbs and their difference (exclusion minus inclusion). **(B)** The topography map for each condition and their difference at the time course from 250 to 500 ms. **(C)** Bar graph showing mean N400 amplitudes at posterior parietal area for inclusion versus exclusion word condition. The asterisk indicates a reliable difference between inclusion and exclusion verbs. Error bars indicate standard error.

EEG recordings (NeuroScan) were taken from 64 tin electrode sites with the reference on the left mastoid and re-referenced off-line to the average of the left and right mastoids. The horizontal electro-oculogram (EOG) was recorded from two additional bipolar electrode sites placed 1 cm lateral to the outer canthi of each eye. The vertical EOG was recorded from electrode sites below and above the left eye. The impedance for all electrode sites was maintained below 5 kΩ. EEG and EOG activities were amplified with a bandpass of 0.01–100 Hz and sampled at 500 Hz/channel. The data were analyzed offline. Ocular artifacts were corrected by NeuroScan software (Semlitsch et al., 1986). Data were filtered with a low-pass filter at 30 Hz (24 dB/octave). The EEG was segmented in epochs of 1000 ms, time-locked to stimuli (the verb words) onset and included a 200 ms pre-stimulus baseline. Trials contaminated by amplifier clipping, bursts of electromyographic activity, or peak-to-peak deflection exceeding ±75 μV were excluded from averaging. According to the grand average ERP waveforms and topographic map (see **Figure 1**), the following nine posterior electrode sites were selected for statistical analysis: left (CP3, P3, PO3); midline (CPz, Pz, POz); right (CP4, P4, PO4). The N400 component was calculated mean amplitudes within 250–500 ms window.

To test whether individuals hold an interpersonal self-positivity bias, we conducted a 2 (verb types: exclusion, inclusion) × 3 (electrode position: left, midline, and right) repeated measures analysis of variance (ANOVA) for N400. To verify our hypothesis, we also examined the correlation between N400 effect at separately posterior positions and loneliness and self-esteem.

## Results

Study statistical power was confirmed by a sensitivity power analysis with G-power3.1.9 software (Faul et al., 2007), to examine what effect size could be detected given the current sample size, and α = 0.05 and a power of 0.80. A correlation among repeated measures of 0.3 and non-sphericity correction of 1 were used based on commonly used values described in Prajapati et al. (2010), the design had the sensitivity to detect an effect of *f* = 0.22, which equaled about 4.62% in explained variance.

Since N400 is a kind of negative going waveform, larger N400 amplitudes corresponded to smaller N400 amplitudes values. The Greenhouse–Geisser correction was used when the assumption of sphericity was violated. The ANOVA revealed a main effect of verb types [*F*(1,30) = 4.99, *p* = 0.03, $\eta_{p}^{2}$ = 0.14]. No Main effect of electrode position [*F*(2,60) = 1.84, *p* = 0.168] and no significant interaction effect [*F*(1.486,44.576) = 2.41, *p* = 0.115, adjusted by Greenhouse–Geisser] were found. Kutas and Federmeier (2011) suggest that N400 has a right hemisphere bias, thus further analysis were conducted and found that exclusion verbs elicited larger N400 amplitudes than inclusion verbs in posterior area, especially at the midline [*F*(1,30) = 6.07, *p* = 0.02, $\eta_{p}^{2}$ = 0.168] at right hemisphere [*F*(1,30) = 5.55, *p* = 0.025, $\eta_{p}^{2}$ = 0.156]; however, they were not significant at left posterior hemisphere [*F*(1,30) = 1.83, *p* = 0.187, $\eta_{p}^{2}$ = 0.057] (see **Figure 1**).

**FIGURE 2:**
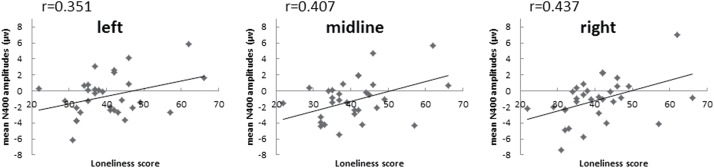
Zero-order correlations between N400 difference amplitudes and loneliness scores at three posterior parietal positions.

N400 effect is indexed by N400 difference amplitudes calculated by exclusion verbs ERP – inclusion verbs ERP. Larger numbers mean a smaller N400 effect [e.g., -10-(-6) = -4, vs. -8-(-7) = -1]. Therefore, the positive correlation between N400 difference and loneliness reflected that with increment of sense of loneliness, N400 effect becomes smaller. In the present experiment, there were significant correlations between N400 difference amplitudes and loneliness scores at midline, right hemisphere of posterior area (*r* = 0.407, *p* = 0.023; *r* = 0.437, *p* = 0.014). The correlation at left posterior hemisphere was marginal (*r* = 0.351, *p* = 0.053) (see **Figure 2**). However, there were no significant correlations between N400 difference amplitudes and SE (*r*_left_ = -0.096, *r*_midline_ = -0.145, *r*_right_ = -0.208), all *p* > 0.2.

To better understand the above correlations, we further analyzed correlations between loneliness scores and N400 of inclusion verbs and N400 of exclusion verbs separately. Results showed that at the right hemisphere of posterior area, there was a weak correlation between mean N400 amplitudes elicited by inclusion verbs and loneliness score (*r* = -0.313, *p* = 0.086), indicating a trend that inclusion verbs trigger larger N400 when individual’s sense of loneliness got higher. However, loneliness scores were not significantly relevant to inclusion N400 at the left or midline posterior area (both *p* > 0.1). Moreover, N400s elicited by exclusion verbs at posterior positions were not correlated with loneliness scores (all *p* > 0.2).

## Discussion

In the present study, we aimed to use the N400 component to seek evidences for interpersonal self-positivity bias and explore how impoverished social interaction affects this bias. By using a modified self-reference task, we found a classic N400 effect with more negative going N400 elicited by exclusion verbs than inclusion verbs. However, with ascent of loneliness, N400 effect became smaller.

### Interpersonal Self-Positivity Bias

N400 is treated as an index sensitive to violation of self-positivity bias (Watson et al., 2007; Zhou et al., 2013; Chen et al., 2014; Metzler et al., 2014; Kiang et al., 2017). In current experiment, we found exclusion verbs elicited larger N400 than inclusion verbs, especially in the midline and right posterior area, which supports our hypothesis that human beings have an interpersonal self-positivity bias. Individuals’ self-concept is constructed, validated and even changed within social interactions (Morf et al., 2011; Richman et al., 2016). Being included by others or groups satisfies human beings’ sense of security and need of belonging. More importantly, it gives individuals a feedback that they are the kind of persons who are likable, social, popular, and valued or respected, which enhances individuals’ good view of themselves. Therefore, interpersonal self-positivity bias is of fundamental significance for most people. Such bias, in turn, promotes people to seek sound social interaction and avoid being excluded by other people. Accordingly, others’ inclusion is taken for granted, which is congruent with individuals’ social self-concept, resulting in smaller N400 amplitudes for inclusion verbs. On the contrary, others’ exclusion is out of expectation, which is incongruent with individuals’ social self-concept, leading to larger N400 amplitudes for exclusion verbs.

By using a lexical decision task, researchers found that female participants produced larger N400 amplitudes for rejection words than acceptance words in partner prime context (Zayas et al., 2009). Zayas et al. (2009) focused on the early automatic-stage processing of cues of partner rejection; however, we recruited a modified self-reference task to explore self-positivity on social interaction dimension. No matter excluded by whom, simply “watching” excluded verbs referenced to self will affect internal cognition, proving that people are sensitive to exclusion cues (Pickett and Gardner, 2005) and have an interpersonal self-positivity bias.

### Loneliness and Interpersonal Self-Positivity Bias

Correlation between loneliness scores and N400 effect showed that N400 effect could be modulated by loneliness, lonely people had smaller interpersonal self-positivity bias. Individuals with high loneliness are characterized with less social interactions quantity and quality, their belonging is chronically threatened, so is their self. The self of lonely people is confused as lonely people cannot get enough feedback information about themselves from social environment and even negative. Our results provided evidence that, for lonely individuals, activation of self-concept were stronger associated with concepts of negative social self to a greater degree than non-lonely individuals. In our study, participants were instructed to process the semantic relationship between self-concept primed “I” and the target verbs. If positively biased interpersonal self-concept is available, then people should expect positive outcomes in sentences related to self than to others (Fields and Kuperberg, 2015). However, Semantic meanings of exclusion verbs remind lonely people of those negative social interaction experiences referenced to themselves, and make them more access to negative outcomes.

We further found that at the right posterior occipital area, loneliness was inclined to be negatively related to N400 amplitudes elicited by inclusion verbs but not to exclusion verbs, which suggested that processing of inclusion information relevant to self might be modulated by loneliness, lonely people produced larger N400 amplitudes for inclusion verbs than non-lonely people. Previous studies showed that lonely people were hyposensitive to inclusion information (Cacioppo et al., 2009; Moller et al., 2010; Vanhalst et al., 2015). As they have experienced less interpersonal relationship in the past, they are generally reinforced to loss expectancies to be included by others (Simpson et al., 2007; Moller et al., 2010). Thus, being included is out of lonely people’s expectancy, violating their negative self-concept in social interaction. Nevertheless, more evidences are required in the future to examine the speculation and make the underlying mechanism clear.

### Exclusivity of Interpersonal Self-Positivity Bias

We regard interpersonal self-positivity bias as a factor or dimension of but different from self-positivity bias. In classic self-reference task, participants respond to adjective traits. By recording neural activities during their response to self-referenced positive and negative traits, researchers found people evaluate themselves positively and hold positive views about themselves. In the present experiment, we employed a modified self-reference task, in which participants were instructed to read verbs referenced to self. Exclusion verbs we selected were rated more negative than inclusion verbs, and were similar in emotional valance as adjectives used in classic self-positivity bias research. Previous studies have found that processing of emotional information is influenced by self-reference (Herbert et al., 2011a,b; Fields and Kuperberg, 2015). Thus, people may doubt that interpersonal self-positivity bias herein is lack of exclusivity. Fortunately, further evidences supported our views.

Lin et al. (2003) once proved that self-positivity bias is a strategic device for self-esteem maintenance. Subsequent studies consistently found people in high self-esteem had more pronounced self-positivity bias; however, those in low self-esteem don’t (Wentura et al., 2005; Tao et al., 2012; Frewen et al., 2013; Zhang et al., 2013). If interpersonal self-positivity bias examined in the present study is the same as self-positivity bias, then N400 effect should be modulated by self-esteem. In fact, we found that loneliness modulated interpersonal self-positivity bias but participants’ self-esteem was not correlated with such bias. That is to say, such two biases are not empirically identical. What we examined is the specific self-positivity modulated by social interaction. People think they are “better than average” and should be included by others. Such interpersonal positivity bias helps people to keep self-confidence in accessing their interpersonal relationship, which is similar to social self-esteem. Nevertheless, self-positivity bias reflects individuals’ general self-evaluation or self-attitude, which is correlated with global self-esteem. Global self-esteem is distinguished from dimension-specific self-esteem (Crocker and Major, 1989). Individuals may be in low social esteem and high in global self-esteem; or evaluate the self positively on a specific dimension but be low in self-esteem (Crocker and Major, 1989). That might be the reason why interpersonal self-positivity bias was not modulated by global self-esteem. Besides, our findings on loneliness gave more details about interpersonal self-positivity bias, which makes it reasonable to speculate that though interpersonal self-positivity bias is a specific dimension of global self-positivity bias, they are not the same.

### Limitations and Future Directions

There are some limitations of the present study. First, the sample size is small, which might account for the small effect size of the study. Additionally, if the sample size was large enough to divide participants into high and low loneliness group, we could examine how high/low lonely individuals process inclusion/exclusion verbs directly. Second, participants were instructed to “watch” words without any responses. As in a typical self-reference task, participants are required to classify traits by button pressing (Zhou et al., 2013; Chen et al., 2014; Metzler et al., 2014; Kiang et al., 2017). Inferences of present study will be greatly enhanced if behavioral data is collected and supports the same conclusions. Third, we cautiously suggested that lonely individuals’ smaller interpersonal self-positivity bias was due to less expectancy of inclusion. As such speculation was based on marginally significant correlation between loneliness and inclusion verbs N400 at the right posterior area. The findings were tentative and more evidences were required. Future studies should replicate the findings depicted here by including a modified task recording behavioral data (e.g., reaction times) and with an enlarged sample size. Moreover, future work can explore how culture interacts with interpersonal self-positivity bias. As Western cultures emphasize independent self and East Asian cultures encourage interconnectedness with surrounding others (Markus and Kitayama, 1991), therefore peoples’ interpersonal self-positivity bias may vary from East Asian culture to Western culture.

## Conclusion

Our study examined interpersonal self-positivity bias at the neurophysiologic level. Exclusion verbs referenced to self-elicited larger N400 amplitudes than inclusion verbs. Further, we found N400 effect was modulated by status of social interaction. Lonely people who are characterized with impoverished social interaction exhibited smaller N400\ effect.

## Ethics Statement

This study was carried out in accordance with the recommendations of local ethics committee of Shanghai Normal University with written informed consent from all subjects in accordance with the Declaration of Helsinki. The protocol was approved by the ethics committee of Shanghai Normal University.

## Author Contributions

MZ, CZ, XG, and JL designed the experiments. MZ and CZ recruited the participants, collected the data, and prepared the draft. MZ performed the data analysis. XG and JL reviewed it critically and gave important intellectual content.

## Conflict of Interest Statement

The authors declare that the research was conducted in the absence of any commercial or financial relationships that could be construed as a potential conflict of interest.
