# Supplementary material for: Lonely Individuals Do Not Show Interpersonal Self-Positivity Bias: Evidence From N400
Source: Front Psychol. 2018 Apr 6;9:473. doi: 10.3389/fpsyg.2018.00473 (PMC5898257; doi:10.3389/fpsyg.2018.00473)
Supplement: Supplementary file 1 [file Data_Sheet_1.docx]

**SUPPLEMENTARY MATERIAL**

Inclusion verbs (in Chinese): 表扬,称赞,喜爱,欣赏,信任,信赖,赞赏,赞美,关爱,赞同,祝福,尊重,理解,接受,认可,关注,支持,鼓励,关心,安慰.

Exclusion verbs (in Chinese): 冷落,疏远,排斥,排挤,拒绝,孤立,抛弃,忽略,忽视,疏离,轻视,怠慢,疏忽,歧视,遗忘,远离,厌恶,嘲弄,嘲笑,无视.
